# Supplementary material for: Italian Translation and Validation of the Original ABC Taxonomy for Medication Adherence
Source: Healthcare (Basel). 2023 Mar 13;11(6):846. doi: 10.3390/healthcare11060846 (PMC10048001; doi:10.3390/healthcare11060846)
Supplement: Supplementary file 1 [file healthcare-11-00846-s001.zip › Supplementary File S1.pdf]

Supplementary Material S1

1 Supplementary Tables

Table S1. Search strategy for conducting Systematic Review of literature

Query number	Terms	Number of records PubMed	Number of records Embase
#1	Medication adherence	32	60
#2	Initiation	24	20
#3	Implementation	231	174
#4	Discontinuation	20	36
#5	Persistence	29	42
#6	Adherence management	0	79
#7	Adherence science	5	5
#8	Adherence	167	164
#9	Compliance	130	209
#10	Patient compliance	39	163
#11	Treatment adherence	5	104
#12	Medication compliance	0	71
#13	Medication persistence	0	13
#14	Treatment compliance	2	134
#15	Adhesion	16	29
#16	Interruption	18	21
#17	1 OR 5 OR 6 OR 7 OR 8 OR 9 OR 10 OR 11 OR 12 OR 13 OR 14 OR 15	297	340
#18	2 OR 3 OR 4 OR 16	289	244
#19	17 AND 18	**30**	**24**

Notes: Language filter: Italian; Time filter: 2012 to 2020.

Table S2. Search strategy – Queries performed

Scientific database	Query performed
**PubMed (Medline)**	(((("Medication adherence"[Mesh]) OR "persistence" OR "adherence management" OR "adherence science" OR "Adherence" OR "compliance" OR "patient compliance" OR "medication compliance" OR "medication persistence" OR "treatment compliance" OR "adhesion") AND ("2012"[Date - Publication] : "2020"[Date - Publication])) AND (("Initiation" OR "Implementation" OR "discontinuation" OR "interruption") AND ("2012"[Date - Publication] : "2020"[Date - Publication])))
**Embase (excl. Medline)**	((('medication'/exp OR medication) AND ('adherence'/exp OR adherence) OR ('persistence' OR (('adherence'/exp OR adherence) AND ('management'/exp OR management) OR (('adherence'/exp OR adherence) AND ('science'/exp OR science) OR (('adherence'/exp OR adherence) OR (('compliance'/exp OR compliance) OR (('patient'/exp OR patient) AND ('compliance'/exp OR compliance)) AND (('treatment'/exp OR treatment) AND ('adherence'/exp OR adherence)) OR (('medication'/exp OR medication) AND ('compliance'/exp OR compliance) OR (('medication'/exp OR medication) AND ('persistence'/exp OR persistence) OR (('treatment'/exp OR treatment) AND ('compliance'/exp OR compliance) OR (('adhesion'/exp OR adhesion)) AND ('initiation' OR 'implementation') OR ('discontinuation' OR (('interruption' OR interruption)) AND [italian]/lim AND [embase]/lim AND [2012-2020]/py)))

Table S3. Scheduling of 7 terms and definitions in italian language

Authors, Year	ABC Taxonomy terms and definitions						
	1. Adherence to medication	2. Initiation	3. Implementation	4. Discontinuation	5. Persistence	6. Management of adherence	7. Adherence-related science
Cillo M.R. et al, 2012[1]	**Aderenza alla terapia/Aderenza al trattamento**	**Inizio della terapia/ Inizio del trattamento**	*Missing*	**Interruzione del trattamento=** si intende un periodo di almeno 60 giorni senza copertura farmacologica	**Persistenza al trattamento=** indica il mantenimento del regime terapeutico nel tempo e quindi, misura per quanti giorni un soggetto dispone della terapia senza potenziali interruzioni.	*Missing*	*Missing*
Angelino E. et al, 2012[2]	**Aderenza alle terapie prescritte/ Aderenza ai farmaci/ Aderenza alle prescrizioni**	*Missing*	Modalità di assunzione corretta dei farmaci	*Missing*	*Missing*	*Missing*	*Missing*
Colonna P. et al, 2013[3]	**Aderenza alla terapia/ Aderenza al trattamento=** ossia l'assunzione continua della terapia nel rispetto delle indicazioni posologiche contenute nelle prescrizioni mediche.	**Inizio della terapia/ Inizio del trattamento**	*Missing*	**Interruzione della terapia/ Interruzione del trattamento**	**Persistenza alla terapia=** ossia la prosecuzione nel tempo del trattamento farmacologico prescritto, senza interruzioni	*Missing*	*Missing*
Schiroli C. et al, 2013[4]	**Adesione del paziente alle terapie; Compliance, Compliance al trattamento**	*Missing*	*Missing*	*Missing*	*Missing*	*Missing*	*Missing*

… continued

Authors, Year	ABC Taxonomy terms and definitions						
	**1. Adherence to medication**	**2. Initiation**	**3. Implementation**	**4. Discontinuation**	**5. Persistence**	**6. Management of adherence**	**7. Adherence-related science**
Greco C. et al, 2014[5]	**Aderenza alla terapia**=comportamento individuale del singolo paziente sostanzialmente privo di una definizione universalmente accettata. I comportamenti anomali tenuti dai pazienti rispetto alla terapia farmacologica.	*Missing*	Assunzione erronea o consapevole di farmaci in dosaggi diversi ed inferiori a quelli prescritti (sottodosaggio)/ Riduzione erronea o consapevole del numero delle somministrazioni dei singoli farmaci	**Vacanza terapeutica=** Omissione parziale o completa della terapia farmacologica per diversi periodi di tempo	**Persistenza terapeutica**	*Missing*	*Missing*
Greco A. et al, 2014[6]	**Compliance al trattamento**	*Missing*	*Missing*	*Missing*	*Missing*	*Missing*	*Missing*
Coluzzi F. et al, 2014[7]	**Aderenza al trattamento**	*Missing*	*Missing*	**Interruzione del trattamento**	*Missing*	*Missing*	*Missing*
Musicco F. et al, 2015[8]	**Aderenza alla terapia/Aderenza al trattamento**	*Missing*	*Missing*	**Interruzione della terapia**	*Missing*	*Missing*	*Missing*
Ravasio R. et al, 2015[9]	**Aderenza al trattamento**	*Missing*	*Missing*	**Interruzione del trattamento**	*Missing*	*Missing*	*Missing*
Gallingani F. et al, 2015[10]	**Aderenza alla terapia**	*Missing*	*Missing*	*Missing*	*Missing*	*Missing*	*Missing*
Minutolo R. et al, 2015[11]	**Aderenza alla terapia**	*Missing*	*Missing*	*Missing*	*Missing*	*Missing*	*Missing*
Pesavento R. et al, 2017[12]	**Aderenza alla terapia**	*Missing*	*Missing*	*Missing*	**Persistenza in terapia**	*Missing*	*Missing*

… continued

Authors, Year	ABC Taxonomy terms and definitions						
	1. Adherence to medication	2. Initiation	3. Implementation	4. Discontinuation	5. Persistence	6. Management of adherence	7. Adherence-related science
Rega M.L. et al, 2018[13]	**Aderenza al trattamento=** il grado o la portatata della conformità alle raccomandazioni relartive al trattamento quitidiano prescritto rispetto al tempo, al dosaggio e alla frequenza	*Missing*	*Missing*	*Missing*	**Persistenza al trattamento=** il periodo di tempo dall'inizio all'interruzione del trattamento ed è un aspetto specifico dell'aderenza.	*Missing*	*Missing*
Dolara A., 2018[14]	**Aderenza alla terapia=** coinvolgimento attivo, volontario e collaborativo del paziente, finalizzato a produrre un risultato terapeutico	*Missing*	*Missing*	**Sospensione/Interruzione**	*Missing*	*Missing*	*Missing*
Font M., 2018[15]	**Aderenza alla terapia/Aderenza terapeutica**	*Missing*	*Missing*	**Interruzione della terapia**	*Missing*	*Missing*	*Missing*
Ferrante F. et al, 2019[16]	*Missing*	**Prima prescrizione / Primo evento occorso/ Inizio della terapia**	*Missing*	**Interruzione della terapia=** Evento calcolato rispetto alla copertura terapeutica dell'ultima prescrizione individuata	**Persistenza della terapia**	*Missing*	*Missing*

… continued

Authors, Year	ABC Taxonomy terms and definitions						
	1. Adherence to medication	2. Initiation	3. Implementation	4. Discontinuation	5. Persistence	6. Management of adherence	7. Adherence-related science
Santoleri F. et al, 2019[17]	**Aderenza al trattamento=** La misura in cui il comportamento di una persona – nell'assumere farmaci, seguire una dieta e/o eseguire cambiamenti nello stile di vita – corrisponde alle raccomandazioni concordate con il personale sanitario	*Missing*	*Missing*	**Interruzione del trattamento/ Interruzione della terapia**	**Persistenza al trattamento=** rappresenta il tempo di utilizzo di un farmaco ed è calcolata come differenza in giorni tra la prima e l'ultima dispensazione	*Missing*	*Missing*
Zerboni G. et al, 2019[18]	**Aderenza allo schema terapeutico/ Aderenza alle terapie**	*Missing*	Episodi in cui la terapia non viene assunta per dimenticanza	Ultime due settimane o il giorno precedente la compilazione del test, o se non è stato assunto in occasione di viaggi/vacanze; l'interruzione volontaria del trattamento sulla base della percezione individuale di benessere/malessere o la difficoltà a seguire rigorosamente uno schema terapeutico prescritto.	*Missing*	*Missing*	*Missing*
Antonione R. et al, 2020[19]	**Aderenza terapeutica**	*Missing*	*Missing*	*Missing*	*Missing*	*Missing*	*Missing*

Table S4. Frequencies of papers citing and/or defining the 7 ABC Taxonomy adherence-related terms.

**7 ABC Taxonomy Terms**	**Number (%) of Papers cited Term**	**Number (%) of Papers explain Definition**
**1. Adherence to medication**	18 (94.7)	5 (26.3)
**2. Initiation**	3 (15.8)	0
**3. Implementation**	0	3 (15.8)
**4. Discontinuation**	10 (52.6)	4 (21.1)
**5. Persistence**	7 (36.8)	4 (21.1)
**6. Management of adherence**	0	0
**7. Adherence-related science**	0	0

Table S5. Types of Italian translations found for each of the 7 adherence-related terms.

**7 ABC Taxonomy Terms**	**Italian Translations**	**N**	**%**
1. Adherence to medication	Aderenza alla terapia	11	61,1
	Aderenza terapeutica	2	11,1
	Aderenza allo schema terapeutico	1	5,6
	Aderenza al trattamento	7	38,9
	Aderenza alla prescrizione	1	5,6
	Aderenza ai farmaci	1	5,6
	Adesione alle terapie	1	5,6
	Compliance	1	5,6
	Compliance al trattamento	1	5,6
2. Initiation	Inizio della terapia	3	100,0
	Inizio del trattamento	2	66,7
	Prima prescrizione	1	33,3
	Primo evento occorso	1	33,3
3. Implementation	-		
4. Discontinuation	Interruzione della terapia	5	50,0
	Interruzione del trattamento	5	50,0
	Interruzione	1	10,0
	Sospensione	1	10,0
	Vacanza terapeutica	1	10,0
5. Persistence	Persistenza alla terapia	3	42,9
	Persistenza al trattamento	3	42,9
	Persistenza terapeutica	1	14,3
6. Management of adherence	-		
7. Adherence-related science	-		

2 Supplementary Figures

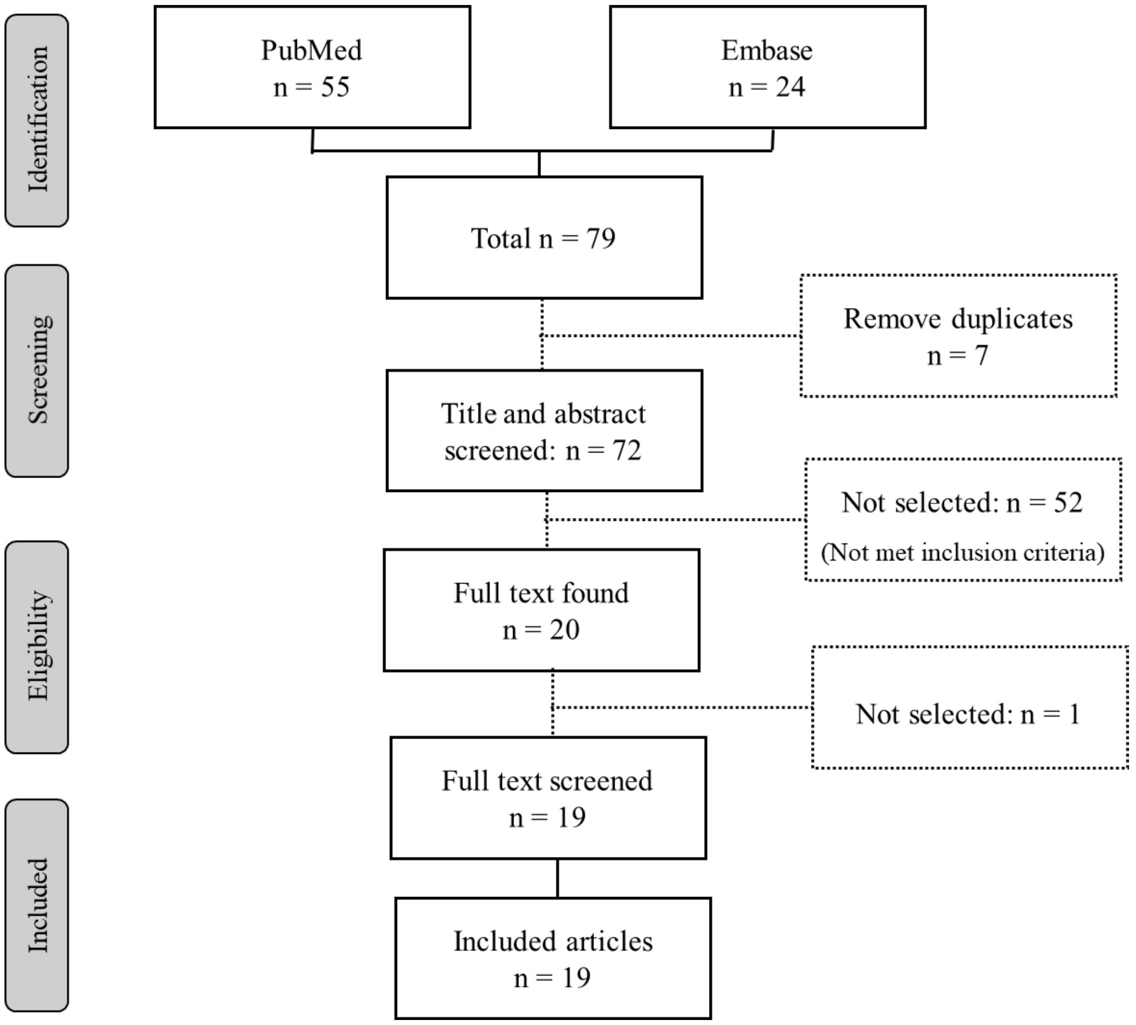

Figure S1. Flow chart.

References

1. Cillo MR, Barra M, Sansone P, Santoni L, Furneri G. L'aderenza alla terapia con statine: un'analisi dai database amministrativi della ASL di Salerno. GIFAC 2012;26(1):31-42.
2. Angelino E, Fattirolli F. L'Informazione essenziale e irrinunciabile alla dimissione dal reparto per acuti dopo sindrome coronarica Parte 1: I fattori che influenzano la comunicazione. Monaldi Arch Chest Dis2012; 78: 79-84.
3. Colonna P, Abrignani MG, Colivicchi F, Verdecchia P, Alunni G, Bongo AS, et al. Documento ANMCO su prevenzione del tromboembolismo nella fibrillazione atriale e ruolo dei nuovi anticoagulanti orali. G Ital Cardiol 2013;14(4):295-322.
4. Schiroli C, Franzetti F. La tubercolosi ricorrente: recidiva o reinfezione esogena? Recurrent tuberculosis: relapse or reinfection? Le Infezioni in Medicina, n. 4, 251-260, 2013.
5. Greco C, Bovenzi FM, Berti S, Abrignani M, Bedogni F, Ceravolo R, et al. Documento ANMCO/GICR-IACPR/GISE. L'organizzazione dell'assistenza nella fase post-acuta delle sindromi coronariche. G Ital Cardiol 2014;15(1 Suppl. 1):3S-27S.
6. Greco A, Scilla CVM, Pugliese R. Verifica del percorso organizzativo di Distribuzione Diretta dell'Ossigenoterapia a Lungo Termine nell'ASP di Catanzaro ambito di Lamezia Terme: appropriatezza prescrittiva e criticità del sistema. GIFAC 2014;28(1):28-40.
7. Coluzzi F, Ruggeri M. Valutazione clinica ed economica di nuovi analgesici per la gestione del dolore cronico. Recenti Prog Med 2014; 105: 415-419.
8. Musicco F, Celeste MG, Mores N, Tufo A, Fraschetti R, Peschi C, Umana E. Valutazione dell'impatto dell'intervento di comunicazione svolto dal farmacista esperto in farmacovigilanza sull'andamento della terapia farmacologica con chemioterapici orali. GIFAC 2015;29(2):84-104.
9. Ravasio R, Nicolò G, Vaggi M. Cost-effectiveness analysis of paliperidone palmitate versus long-acting risperidone as maintenance treatment in schizophrenia patients in Italy. Vol 2 No 1 (2015): January-April 2015.
10. Gallingani F, Piccinni C, Simeoni A, Poluzzi E, Menchetti M, Berardi D. La centralità del paziente nella terapia antipsicotica: condivisione del trattamento, aderenza e ricerca. Recenti Prog Med 2015; 106(11):551-563.
11. Minutolo R, Liberti ME, Garofalo C, Pacilio M, Sagliocchi A, Sguazzo A, et al. Ferro carbossimaltosio nella malattia renale cronica non dialitica: una iniziale esperienza clinica. G Ital Nefrol 2015; 32 (5) – ISSN 1724-5590.
12. Pesavento R, Iori I. Esperienza clinica con rivaroxaban nel trattamento del tromboembolismo venoso: risultati dell'indagine epidemiologica italiana Survey TEV. G Ital Cardiol 2017;18(3):239-246.
13. Rega ML, Calabrese C, Tortorella P, de Waure C, Damiani G. The Adherence's indicators of cancer patients to oral chemotherapy. A sistematic literature review. Prof Inferm. Apr-Jun 2018;71(2):67-78.
14. Dolara A. Aderenza alla terapia farmacologica nelle malattie cardiovascolari. Recenti Prog Med 2018;109(2):108-112.
15. Font M. Alcune considerazioni sull'aderenza alla terapia. Recenti Prog Med 2018;109(2):95-97.
16. Ferrante F, Bianchi C, Iadecola A. Analisi della persistenza della terapia farmacologica: uso di NAO/AVK per pazienti con fibrillazione atriale non valvolare. Studio di coorte su database amministrativi. GIFAC 2019;33(2):46-57.
17. Santoleri F, Lasala R, Abrate P, Musicco F, Costantini A. La Real World Data e la Farmacoutilizzazione: strumenti per il farmacista ospedaliero. GIFAC 2019;33(2):77-85.
18. Zerboni G, Festa S, Aratari A, d'Alessio F, Viscido A, Papi C. L'importanza del coinvolgimento attivo del paziente nella gestione delle malattie infiammatorie croniche dell'intestino. Recenti Prog Med 2019;110(5):236-243.
19. Antonione R, Fieramosca M, Moroni M, Nodari S, Valle A. Overview and symptom management in heart failure patients eligible for palliative care. G Ital Cardiol (Rome). 2020 Aug;21(8):629-638.
