# Supplementary material for: Italian Translation and Validation of the Original ABC Taxonomy for Medication Adherence
Source: Healthcare (Basel). 2023 Mar 13;11(6):846. doi: 10.3390/healthcare11060846 (PMC10048001; doi:10.3390/healthcare11060846)
Supplement: Supplementary file 1 [file healthcare-11-00846-s001.zip › Supplementary File S3.pdf]

## Supplementary Material S3

**ROUND-1 DELPHI SURVEY**

**Selezioni il suo ambito professionale:**

- Biologia
- Biostatistica
- Economia/gestione della salute
- Infermieristica
- Medicina
- Psicologia
- Scienze farmaceutiche
- Scienze sociali
- Altre (specificare:…)

**In ottemperanza alle norme sulla privacy, i dati raccolti saranno usati solo per finalità di ricerca ed in forma aggregata. Acconsente al trattamento dei dati?**

- Acconsento
- Non acconsento

**Primo Termine della tassonomia ABC: "Adherence to medication". Si prega di scegliere UNA SOLA opzione, che secondo lei traduca meglio il termine in italiano:**

- Aderenza alla terapia farmacologica
- Aderenza alla terapia
- Aderenza terapeutica
- Aderenza al trattamento farmacologico
- Aderenza alle prescrizioni farmacologiche
- Aderenza allo schema terapeutico
- Adesione alle terapie farmacologiche
- Aderenza ai farmaci
- Aderenza alle raccomandazioni
- Nessuna delle precedenti

**Suggerite un'altra traduzione per questo termine?**

*Open text...*

**Definizione di "Adherence to medication": "The process by which patients take their medications as prescribed". Si prega di scegliere UNA SOLA opzione, che secondo lei traduce meglio in italiano la descrizione del termine:**

- Il processo secondo cui i pazienti prendono i loro farmaci come prescritto.
- Il processo attraverso cui i pazienti assumono i loro trattamenti come prescritti.
- La misura in cui il comportamento di una persona nell'assumere farmaci corrisponde alle raccomandazioni concordate con il personale sanitario.
- Il grado o la portata della conformità alle raccomandazioni relative al trattamento farmacologico quotidiano prescritto rispetto al tempo, al dosaggio e alla frequenza.
- L'assunzione continua della terapia nel rispetto delle indicazioni posologiche contenute nelle prescrizioni mediche.
- Il comportamento individuale del singolo paziente rispetto alla terapia farmacologica

- Nessuna delle precedenti

**Suggerite un'altra traduzione per questo termine?**

*Open text...*

**Secondo Termine della tassonomia ABC: “Initiation”. Si prega di scegliere UNA SOLA opzione, che secondo lei traduce meglio il termine in italiano:**

- Inizio del trattamento farmacologico
- Inizio della terapia farmacologica
- Prima prescrizione farmacologica
- Primo evento occorso
- Nessuna delle precedenti

**Suggerite un'altra traduzione per questo termine?**

*Open text...*

**Definizione di “Initiation”: “The process starts with initiation of the treatment, when the patient takes the first dose of a prescribed medication”. Si prega di scegliere UNA SOLA opzione, che secondo lei traduce meglio in italiano:**

- Il processo inizia con l'inizio del trattamento, quando il paziente assume la prima dose di un farmaco prescritto.
- Il processo inizia quando il paziente assume la prima dose del farmaco prescrittogli.
- Iniziazione è un processo che si sviluppa con l’inizio del trattamento quando il paziente prende la prima dose del trattamento stesso.
- Nessuna delle precedenti

**Suggerite un'altra traduzione per questo termine?**

*Open text...*

**Terzo Termine della tassonomia ABC: “Implementation”. Si prega di scegliere UNA SOLA opzione, che secondo lei traduce meglio il termine in italiano:**

- Effettiva assunzione della terapia al dosaggio prescritto
- Andare a regime di dosaggio prescritto
- Implementazione
- Attuazione della terapia prescritta
- Messa in atto della terapia prescritta
- Nessuna delle precedenti

**Suggerite un'altra traduzione per questo termine?**

*Open text...*

**Definizione di “Implementation”: “The process continues with the implementation of the dosing regimen, defined as the extent to which a patient’s actual dosing corresponds to the prescribed dosing regimen, from initiation until the last dose is taken”. Si prega di scegliere UNA SOLA opzione, che secondo lei traduce meglio in italiano:**

- Il processo continua con il raggiungimento del regime di dosaggio farmacologico prescritto, definito come la misura in cui il dosaggio effettivamente assunto dal paziente corrisponde a quello prescrittogli, dall’inizio della terapia fino all’assunzione dell’ultima dose.
- Il processo continua con l'implementazione del regime di dosaggio, definito come la misura in cui il dosaggio effettivo di un paziente corrisponde al regime di dosaggio prescritto, dall'inizio fino all'assunzione dell'ultima dose.
- Il processo di realizzazione del trattamento da parte del paziente del regime del dosaggio definito come la misura con cui il dosaggio effettivamente assunto corrisponde al dosaggio prescritto, dall’inizio fino all’assunzione dell’ultima dose.
- Modalità di assunzione corretta dei farmaci.
- Nessuna delle precedenti

**Suggerite un'altra traduzione per questo termine?**

*Open text...*

**Quarto Termine della tassonomia ABC: "Discontinuation". Si prega di scegliere UNA SOLA opzione, che secondo lei traduce meglio il termine in italiano:**

- Interruzione della terapia farmacologica
- Interruzione del trattamento farmacologico
- Sospensione della terapia farmacologica
- Interruzione
- Vacanza terapeutica
- Nessuna delle precedenti

**Suggerite un'altra traduzione per questo termine?**

*Open text...*

**Definizione di "Discontinuation": "Discontinuation marks the end of therapy, when the next dose to be taken is omitted and no more doses are taken thereafter". Si prega di scegliere UNA SOLA opzione, che secondo lei traduce meglio in italiano:**

- L'interruzione definisce la fine della terapia, quando la dose successiva da assumere viene omessa e non vengono più assunte altre dosi.
- La sospensione segna la fine della terapia e si verifica quando il paziente omette la dose successiva e tutte le seguenti, senza alcun accordo con il medico prescrittore.
- Omissione parziale o completa della terapia farmacologica per diversi periodi di tempo.
- Si intende un periodo di giorni senza copertura farmacologica.
- Evento calcolato rispetto alla copertura terapeutica dell'ultima prescrizione individuata.
- Nessuna delle precedenti

**Suggerite un'altra traduzione per questo termine?**

*Open text...*

**Quinto Termine della tassonomia ABC: "Persistence". Si prega di scegliere UNA SOLA opzione, che secondo lei traduce meglio il termine in italiano:**

- Persistenza al trattamento farmacologico
- Persistenza alla terapia farmacologica
- Persistenza terapeutica
- Nessuna delle precedenti

**Suggerite un'altra traduzione per questo termine?**

*Open text...*

**Definizione di "Persistence": "Persistence is the length of time between initiation and the last dose, which immediately precedes discontinuation". Si prega di scegliere UNA SOLA opzione, che secondo lei traduce meglio in italiano:**

- La persistenza è il tempo che intercorre tra l'inizio e l'ultima dose, che precede immediatamente l'interruzione.
- La persistenza è il periodo di tempo tra l'inizio della terapia e l'ultima dose che precede immediatamente la sospensione.
- Indica il mantenimento del regime terapeutico nel tempo e quindi, misura per quanti giorni un soggetto dispone della terapia senza potenziali interruzioni.
- La prosecuzione nel tempo del trattamento farmacologico prescritto, senza interruzioni.
- Rappresenta il tempo di utilizzo di un farmaco ed è calcolata come differenza in giorni tra la prima e l'ultima dispensazione.
- Il periodo di tempo dall'inizio all'interruzione del trattamento ed è un aspetto specifico dell'aderenza.
- Nessuna delle precedenti

**Suggerite un'altra traduzione per questo termine?**

*Open text...*

**Sesto Termine della tassonomia ABC: "Management of adherence". Si prega di scegliere UNA SOLA opzione, che secondo lei traduce meglio il termine in italiano:**

- Gestione dell'aderenza terapeutica
- Gestione dell'aderenza
- Nessuna delle precedenti

**Suggerite un'altra traduzione per questo termine?**

*Open text...*

**Definizione di "Management of adherence": "It is the process of monitoring and supporting patients' adherence to medications by health care systems, providers, patient, and their social networks. The objective of management of adherence is to achieve the best use by patients, of appropriately prescribed medicines, in order to maximise the potential for benefit and minimise the risk of harm". Si prega di scegliere UNA SOLA opzione, che secondo lei traduce meglio in italiano:**

- È il processo di monitoraggio e sostegno all'aderenza dei pazienti al trattamento da parte dei sistemi e degli operatori sanitari, dei pazienti e delle loro reti sociali. L'obiettivo della gestione dell'aderenza è quello di ottenere da parte dei pazienti il miglior utilizzo possibile dei farmaci adeguatamente prescritti, al fine di rendere massimo il potenziale di beneficio e minimo il rischio di danno.
- È il processo di monitoraggio e di sostegno dell'aderenza al trattamento dei pazienti da parte dei sistemi sanitari, dei fornitori, del paziente e dei loro social network. L'obiettivo della gestione dell'aderenza è quello di ottenere il miglior uso possibile da parte dei pazienti, dei farmaci opportunamente prescritti, al fine di massimizzare il potenziale di beneficio e ridurre al minimo il rischio di danni.
- Nessuna delle precedenti

**Suggerite un'altra traduzione per questo termine?**

*Open text...*

**Settimo Termine della tassonomia ABC: "Adherence-related science". Si prega di scegliere UNA SOLA opzione, che secondo lei traduce meglio il termine in italiano:**

- Scienza rivolta allo studio dell'aderenza
- Scienza dell'aderenza terapeutica
- Scienza correlata all'aderenza
- Nessuna delle precedenti

**Suggerite un'altra traduzione per questo termine?**

*Open text...*

**Definizione di "Adherence-related science": "This element includes the disciplines that seek understanding of the causes or consequences of differences between the prescribed (i.e. intended) and actual exposures to medicines. The complexity of this field, as well as its richness, results from the fact that it operates across the boundaries between many disciplines, including but not limited to medicine, pharmacy, nursing, behavioural science, sociology, pharmacometrics, biostatistics and health economics". Si prega di scegliere UNA SOLA opzione, che secondo lei traduce meglio in italiano:**

- Questo elemento include le discipline che mirano a comprendere le cause o le conseguenze della differenza tra l'esposizione prescritta ai farmaci (cioè prevista dal medico prescrittore) e l'esposizione effettiva. La complessità di questo campo di ricerca, così come la sua ricchezza, derivano dal fatto che esso opera oltre i confini di diverse discipline, tra le quali, ma non solo, la medicina, la farmacia, le scienze infermieristiche, le scienze comportamentali, la sociologia, la farmacometria, la biostatistica e l'economia sanitaria.
- Questo elemento comprende le discipline che cercano di comprendere le cause o le conseguenze delle differenze tra le esposizioni ai farmaci prescritte (cioè previste) e quelle effettive. La complessità di questo campo, così come la sua ricchezza, deriva dal fatto che esso opera oltre i confini tra molte discipline, tra cui, ma non solo, la medicina, la farmacia, l'infermieristica, le scienze comportamentali, la sociologia, la farmacometria, la biostatistica e l'economia sanitaria.
- Nessuna delle precedenti

**Suggerite un'altra traduzione per questo termine?**

*Open text...*

**ROUND-2 DELPHI SURVEY**

**Primo Termine della tassonomia ABC: "Adherence to medication". Si prega di scegliere UNA SOLA opzione, che secondo lei traduca meglio il termine in italiano:**

- Aderenza alla terapia farmacologica
- Aderenza alla terapia

**Suggerisce un'altra traduzione per questo termine?**

*Open text…*

**Definizione di "Adherence to medication": "The process by which patients take their medications as prescribed". Si prega di scegliere UNA SOLA opzione, che secondo lei traduce meglio in italiano la descrizione del termine:**

- Il processo attraverso cui i pazienti assumono i loro trattamenti come prescritto.
- La misura in cui il comportamento di una persona nell'assumere le terapie prescritte corrisponde alle raccomandazioni concordate con il personale sanitario.
- Il processo secondo cui i pazienti prendono i loro farmaci come prescritto.

**Suggerisce un'altra traduzione per questo termine?**

*Open text…*

**Secondo Termine della tassonomia ABC: "Initiation". Si prega di scegliere UNA SOLA opzione, che secondo lei traduce meglio il termine in italiano:**

- Inizio della terapia farmacologica
- Inizio del trattamento farmacologico

**Suggerisce un'altra traduzione per questo termine?**

*Open text…*

**Definizione di "Initiation": "The process starts with initiation of the treatment, when the patient takes the first dose of a prescribed medication". Si prega di scegliere UNA SOLA opzione, che secondo lei traduce meglio in italiano:**

- Il processo inizia con l'inizio del trattamento, quando il paziente assume la prima dose di un farmaco prescritto.
- Il processo inizia quando il paziente assume la prima dose del farmaco prescrittogli.

**Suggerisce un'altra traduzione per questa definizione?**

*Open text…*

**Terzo Termine della tassonomia ABC: "Implementation". Si prega di scegliere UNA SOLA opzione, che secondo lei traduce meglio il termine in italiano:**

- Effettiva assunzione della terapia al dosaggio prescritto
- Andare a regime di dosaggio prescritto
- Implementazione

**Suggerisce un'altra traduzione per questo termine?**

*Open text…*

**Definizione di "Implementation": "The process continues with the implementation of the dosing regimen, defined as the extent to which a patient's actual dosing corresponds to the prescribed dosing regimen, from initiation until the last dose is taken". Si prega di scegliere UNA SOLA opzione, che secondo lei traduce meglio in italiano:**

- Il processo continua con il raggiungimento del regime di dosaggio farmacologico prescritto, definito come la misura in cui il dosaggio effettivamente assunto dal paziente corrisponde a quello prescrittogli, dall'inizio della terapia fino all'assunzione dell'ultima dose.
- Il processo continua con l'implementazione del regime di dosaggio, definito come la misura in cui il dosaggio effettivo di un paziente corrisponde al regime di dosaggio prescritto, dall'inizio fino all'assunzione dell'ultima dose.

**Suggerisce un'altra traduzione per questa definizione?**

*Open text…*

**Quarto Termine della tassonomia ABC: "Discontinuation". Si prega di scegliere UNA SOLA opzione, che secondo lei traduce meglio il termine in italiano:**

- Interruzione della terapia farmacologica
- Interruzione del trattamento farmacologico

**Suggerisce un'altra traduzione per questo termine?**

*Open text…*

**Definizione di "Discontinuation": "Discontinuation marks the end of therapy, when the next dose to be taken is omitted and no more doses are taken thereafter". Si prega di scegliere UNA SOLA opzione, che secondo lei traduce meglio in italiano:**

- L'interruzione definisce la fine della terapia, quando la dose successiva da assumere viene omessa e non vengono più assunte altre dosi.
- La sospensione segna la fine della terapia e si verifica quando il paziente omette la dose successiva e tutte le seguenti, senza alcun accordo con il medico prescrittore.

**Suggerisce un'altra traduzione per questa definizione?**

*Open text…*

**Quinto Termine della tassonomia ABC: "Persistence". Si prega di scegliere UNA SOLA opzione, che secondo lei traduce meglio il termine in italiano:**

- Persistenza alla terapia farmacologica
- Persistenza al trattamento farmacologico

**Suggerisce un'altra traduzione per questo termine?**

*Open text…*

**Definizione di "Persistence": "Persistence is the length of time between initiation and the last dose, which immediately precedes discontinuation". Si prega di scegliere UNA SOLA opzione, che secondo lei traduce meglio in italiano:**

- La persistenza è il periodo di tempo tra l'inizio della terapia e l'ultima dose che precede immediatamente la sospensione.
- La persistenza è il tempo che intercorre tra l'inizio e l'ultima dose immediatamente precedente l'interruzione.

**Suggerisce un'altra traduzione per questa definizione?**

*Open text…*

**Sesto Termine della tassonomia ABC: "Management of adherence". Si prega di scegliere UNA SOLA opzione, che secondo lei traduce meglio il termine in italiano:**

- Gestione dell'aderenza terapeutica
- Gestione dell'aderenza

**Suggerisce un'altra traduzione per questo termine?**

*Open text…*

**Definizione di "Management of adherence": "It is the process of monitoring and supporting patients' adherence to medications by health care systems, providers, patient, and their social networks. The objective of management of adherence is to achieve the best use by patients, of appropriately prescribed medicines, in order to maximise the potential for benefit and minimise the risk of harm". Si prega di scegliere UNA SOLA opzione, che secondo lei traduce meglio in italiano:**

- È il processo di monitoraggio e sostegno all'aderenza dei pazienti al trattamento da parte dei sistemi e degli operatori sanitari, dei pazienti e delle loro reti sociali. L'obiettivo della gestione dell'aderenza è quello di ottenere da parte dei pazienti il miglior utilizzo possibile dei farmaci adeguatamente prescritti, al fine di rendere massimo il potenziale di beneficio e minimo il rischio di danno.
- È il processo di monitoraggio e di sostegno dell'aderenza al trattamento dei pazienti da parte dei sistemi sanitari, dei fornitori, del paziente e dei loro social network. L'obiettivo della gestione dell'aderenza è quello di ottenere il miglior uso possibile da parte dei pazienti, dei farmaci opportunamente prescritti, al fine di massimizzare il potenziale di beneficio e ridurre al minimo il rischio di danni.

**Suggerisce un'altra traduzione per questa definizione?**

*Open text…*

**Settimo Termine della tassonomia ABC: "Adherence-related science". Si prega di scegliere UNA SOLA opzione, che secondo lei traduce meglio il termine in italiano:**

- Scienza rivolta allo studio dell'aderenza
- Scienza dell'aderenza terapeutica
- Scienza correlata all'aderenza

**Suggerisce un'altra traduzione per questo termine?**

*Open text…*

**Definizione di "Adherence-related science": "This element includes the disciplines that seek understanding of the causes or consequences of differences between the prescribed (i.e. intended) and actual exposures to medicines. The complexity of this field, as well as its richness, results from the fact that it operates across the boundaries between many disciplines, including but not limited to medicine, pharmacy, nursing, behavioural science, sociology, pharmacometrics, biostatistics and health economics". Si prega di scegliere UNA SOLA opzione, che secondo lei traduce meglio in italiano:**

- Questo elemento include le discipline che mirano a comprendere le cause o le conseguenze della differenza tra l'esposizione prescritta ai farmaci (cioè prevista dal medico prescrittore) e l'esposizione effettiva. La complessità di questo campo di ricerca, così come la sua ricchezza, derivano dal fatto che esso opera oltre i confini di diverse discipline, tra le quali, ma non solo, la medicina, la farmacia, le scienze infermieristiche, le scienze comportamentali, la sociologia, la farmacometria, la biostatistica e l'economia sanitaria.
- Questo elemento comprende le discipline che cercano di comprendere le cause o le conseguenze delle differenze tra le esposizioni ai farmaci prescritte (cioè previste) e quelle effettive. La complessità di questo campo, così come la sua ricchezza, deriva dal fatto che esso opera oltre i confini tra molte discipline, tra cui, ma non solo, la medicina, la farmacia, l'infermieristica, le scienze comportamentali, la sociologia, la farmacometria, la biostatistica e l'economia sanitaria.

**Suggerisce un'altra traduzione per questa definizione?**

*Open text…*

## ROUND-3 DELPHI SURVEY

**Primo Termine della tassonomia ABC: "Adherence to medication". Si prega di scegliere UNA SOLA opzione, che secondo lei traduca meglio il termine in italiano:**

- Aderenza alla terapia farmacologica
- Aderenza alla terapia

**Suggerite un'altra traduzione per questo termine?**

*Open text…*

**Definizione di "Adherence to medication": "The process by which patients take their medications as prescribed". Si prega di scegliere UNA SOLA opzione, che secondo lei traduce meglio in italiano la descrizione del termine:**

- Il processo attraverso cui i pazienti assumono i loro farmaci come prescritto.
- La misura in cui il comportamento di una persona nell'assumere le terapie prescritte corrisponde alle raccomandazioni concordate con il personale sanitario.

**Suggerisce un'altra traduzione per questa definizione?**

*Open text…*

**Secondo Termine della tassonomia ABC: "Initiation". Si prega di scegliere UNA SOLA opzione, che secondo lei traduce meglio il termine in italiano:**

- Inizio della terapia farmacologica
- Inizio del trattamento farmacologico

**Suggerisce un'altra traduzione per questo termine?**

*Open text…*

**Definizione di “Initiation”: “The process starts with initiation of the treatment, when the patient takes the first dose of a prescribed medication”. Si prega di scegliere UNA SOLA opzione, che secondo lei traduce meglio in italiano:**

- Il processo inizia con l'inizio del trattamento, quando il paziente assume la prima dose di un farmaco prescritto.
- Il processo inizia quando il paziente assume la prima dose del farmaco prescrittogli.

**Suggerisce un'altra traduzione per questo termine?**

*Open text…*

**Terzo Termine della tassonomia ABC: “Implementation”. Si prega di scegliere UNA SOLA opzione, che secondo lei traduce meglio il termine in italiano:**

- Effettiva assunzione della terapia al dosaggio prescritto
- Implementazione del trattamento farmacologico

**Suggerisce un'altra traduzione per questo termine?**

*Open text…*

**Definizione di “Implementation”: “The process continues with the implementation of the dosing regimen, defined as the extent to which a patient’s actual dosing corresponds to the prescribed dosing regimen, from initiation until the last dose is taken”. Si prega di scegliere UNA SOLA opzione, che secondo lei traduce meglio in italiano:**

- Il processo continua con il raggiungimento del regime di dosaggio farmacologico prescritto, definito come la misura in cui il dosaggio effettivamente assunto dal paziente corrisponde a quello prescrittogli, dall’inizio della terapia fino all’assunzione dell’ultima dose.
- Il processo continua con l'implementazione del regime terapeutico, definito come il grado di concordanza tra il dosaggio effettivamente assunto dal paziente e il dosaggio prescritto, dall'inizio della terapia fino all'assunzione dell'ultima dose.

**Suggerisce un'altra traduzione per questo termine?**

*Open text…*

**Quarto Termine della tassonomia ABC: “Discontinuation”. Si prega di scegliere UNA SOLA opzione, che secondo lei traduce meglio il termine in italiano:**

- Interruzione della terapia farmacologica
- Interruzione del trattamento farmacologico

**Suggerisce un'altra traduzione per questo termine?**

*Open text…*

**Definizione di “Discontinuation”: “Discontinuation marks the end of therapy, when the next dose to be taken is omitted and no more doses are taken thereafter”. Si prega di scegliere UNA SOLA opzione, che secondo lei traduce meglio in italiano:**

- L'interruzione definisce la fine della terapia, quando la dose successiva da assumere viene omessa e non vengono più assunte altre dosi.
- L’interruzione definisce la fine della terapia e si verifica quando il paziente omette la dose successiva e tutte le seguenti, senza alcun accordo con il medico prescrittore.

**Suggerisce un'altra traduzione per questo termine?**

*Open text…*

**Quinto Termine della tassonomia ABC: “Persistence”. Si prega di scegliere UNA SOLA opzione, che secondo lei traduce meglio il termine in italiano:**

- Persistenza alla terapia farmacologica
- Persistenza al trattamento farmacologico

**Suggerisce un'altra traduzione per questo termine?**

*Open text…*

**Definizione di “Persistence”: “Persistence is the length of time between initiation and the last dose, which immediately precedes discontinuation”. Si prega di scegliere UNA SOLA opzione, che secondo lei traduce meglio in italiano:**

- La persistenza è il periodo di tempo tra l’inizio della terapia e l’ultima dose assunta immediatamente precedente l’interruzione.
- La persistenza è il tempo che intercorre tra l'inizio e l'ultima dose immediatamente precedente l'interruzione.

**Suggerisce un'altra traduzione per questo termine?**

*Open text…*

**Sesto Termine della tassonomia ABC: “Management of adherence”. Si prega di scegliere UNA SOLA opzione, che secondo lei traduce meglio il termine in italiano:**

- Gestione dell’aderenza terapeutica
- Gestione dell'aderenza

**Suggerisce un'altra traduzione per questo termine?**

*Open text…*

**Definizione di “Management of adherence”: “It is the process of monitoring and supporting patients' adherence to medications by health care systems, providers, patient, and their social networks. The objective of management of adherence is to achieve the best use by patients, of appropriately prescribed medicines, in order to maximise the potential for benefit and minimise the risk of harm”. Si prega di scegliere UNA SOLA opzione, che secondo lei traduce meglio in italiano:**

- È il processo di monitoraggio e sostegno dell’aderenza alla terapia dei pazienti da parte dei sistemi e degli operatori sanitari, dei pazienti e delle loro reti sociali. L’obiettivo della gestione dell’aderenza è quello di ottenere, da parte dei pazienti, il miglior utilizzo possibile dei farmaci adeguatamente prescritti, al fine di rendere massimo il beneficio e minimo il rischio di danno.
- È il processo di monitoraggio e di sostegno dell'aderenza al trattamento dei pazienti da parte dei sistemi sanitari, dei fornitori, del paziente e dei loro social network. L'obiettivo della gestione dell'aderenza è quello di ottenere il miglior uso possibile da parte dei pazienti, dei farmaci opportunamente prescritti, al fine di massimizzare il potenziale di beneficio e ridurre al minimo il rischio di danni.

**Suggerisce un'altra traduzione per questo termine?**

*Open text…*

**Settimo Termine della tassonomia ABC: “Adherence-related science”. Si prega di scegliere UNA SOLA opzione, che secondo lei traduce meglio il termine in italiano:**

- Scienza rivolta allo studio dell’aderenza
- Scienza dell’aderenza terapeutica

**Suggerisce un'altra traduzione per questo termine?**

*Open text…*

**Definizione di “Adherence-related science”: “This element includes the disciplines that seek understanding of the causes or consequences of differences between the prescribed (i.e. intended) and actual exposures to medicines. The complexity of this field, as well as its richness, results from the fact that it operates across the boundaries between many disciplines, including but not limited to medicine, pharmacy, nursing, behavioural science, sociology, pharmacometrics, biostatistics and health economics”. Si prega di scegliere UNA SOLA opzione, che secondo lei traduce meglio in italiano:**

- Questo elemento include le discipline che mirano a comprendere le cause o le conseguenze della differenza tra l’esposizione prescritta (cioè prevista dal medico prescrittore) e l’esposizione effettiva. La complessità di questo campo di ricerca, così come la sua ricchezza, derivano dal fatto che esso opera al confine tra diverse discipline, tra le quali figurano, ma non solo, medicina, farmacia, scienze infermieristiche, scienze comportamentali, sociologia, farmacometria, biostatistica, economia sanitaria.
- Questo elemento comprende le discipline che cercano di comprendere le cause o le conseguenze delle differenze tra le esposizioni ai farmaci prescritte (cioè previste) e quelle effettive. La complessità di questo campo, così come la sua ricchezza, deriva dal fatto che esso opera oltre i confini tra molte discipline, tra cui, ma non solo, la medicina, la farmacia, l'infermieristica, le scienze comportamentali, la sociologia, la farmacometria, la biostatistica e l'economia sanitaria.

**Suggerisce un'altra traduzione per questo termine?**

*Open text…*
